# The FOXO’s Advantages of Being a Family: Considerations on Function and Evolution

**DOI:** 10.3390/cells9030787

**Published:** 2020-03-24

**Authors:** Michel Schmitt-Ney

**Affiliations:** Molecular Biotechnology Center, University of Torino, Via Nizza 52, 10126 Torino, Italy; michel.schmitt-ney@unito.it

**Keywords:** FOXO, transcription, gene family, PI3K, AKT, insulin pathway, tumor suppressor, cancer, aging, gene duplication, genetic redundancy, evolution, FOX, forkhead, metabolism

## Abstract

The nematode *Caenorhabditis elegans* possesses a unique (with various isoforms) FOXO transcription factor DAF-16, which is notorious for its role in aging and its regulation by the insulin-PI3K-AKT pathway. In humans, five genes (including a protein-coding pseudogene) encode for FOXO transcription factors that are targeted by the PI3K-AKT axis, such as in *C. elegans*. This common regulation and highly conserved DNA-binding domain are the pillars of this family. In this review, I will discuss the possible meaning of possessing a group of very similar proteins and how it can generate additional functionality to more complex organisms. I frame this discussion in relation to the much larger super family of Forkhead proteins to which they belong. FOXO members are very often co-expressed in the same cell type. The overlap of function and expression creates a certain redundancy that might be a safeguard against the accidental loss of FOXO function, which could otherwise lead to disease, particularly, cancer. This is one of the points that will be examined in this “family affair” report.

## 1. Introduction

Gene duplication is one of the mechanisms that generate functional novelty in evolution. One of the two paralogs created by genomic duplication escapes the pressure of having to maintain its functionality (which is covered by its sibling paralog) and can therefore evolve and diversify toward a new functionality. This concept was initially formulated by Ohno in 1970 [1,2], is largely accepted and has been shown to be a fundamental mechanism for the generation of gene and functional diversity. Progress in sequencing has allowed the evolutionary outcome of duplication to be examined in more detail. Emerging contradictions have helped to reveal the difficulties inherent in understanding the evolutionary forces involved [3].

It would be intuitive to think that the degree to which the two paralogs differ will determine the degree of novelty of the new functionality and that the more important the variations are, the more functional novelty will be created. However, it could be that the creation of functional novelty is dependent on the fact that paralogs remain very similar and this because of the peculiar mode of action of the gene product involved in duplication. This assumption, which, at first may sound paradoxical, may be one of the reasons for the existence, in a given genome, of gene families that encode for very similar proteins. The FOXO family of transcription factors that is present in humans is one such family. In this review, I will discuss the functional implications of the existence of a restricted family of very similar proteins like the FOXOs, but also part of a more extended family of related FOX factors that most probably evolved from a common ancestor.

## 2. FOX Super Family

**Figure 1:**
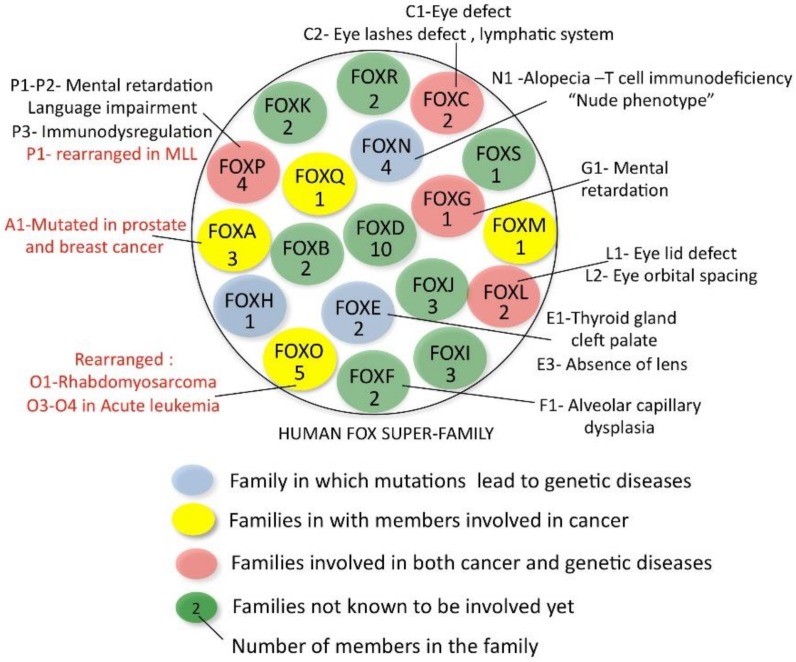
A schematic representation of the human FOX family of transcription factors. The members involved in genetic diseases and cancer are indicated. The arrangement of the families is independent from functional or evolution considerations.

Figure 1 shows a schematic representation of the 19 families (FOXA-FOXS) that make up the FOX super family [4,5]. Each family is composed of 1 to 5 members, and, with a total of about 50 proteins in humans, the FOX super family is the fourth largest transcription factor super family after the homeodomain, bHLH and bZIP families [6]. All FOX members share their very similar forkhead DNA-binding domains, named after the forkhead phenotype that was observed in *Drosophila* and caused by the mutation in one of the precursors of this family [7]. This domain is about 100 amino-acids long and is also called “winged helix” because of the structure it adopts (three alpha-helices, three beta sheets, the last of which is flanked by two loops or wings) [8,9].

Subfamilies are characterized by additional similarities in the forkhead domain but mainly outside of the forkhead domain [10].

FOX members belong to the functional group of transcription factors that are able to bind DNA in a sequence-specific way, on promoters and enhancers, to regulate gene expression. The standard view of a transcription factor binding to a specific DNA sequence and, via this process, recruiting co-factors to influence transcription is by no way wrong, but it represents only part of how transcription factors actually work. Recent studies that used new genome-wide technologies have enlarged our view about sequence-specific DNA recognition in the context of chromatin and about additional mechanisms involved [6,11,12].

FOX proteins participate in the most diverse range of biological functions [10,13,14,15] and are often found to be involved in human diseases, making their study even more interesting. A number of families whose members are responsible for genetic diseases or cancers in humans are indicated in Figure 1. *FOXO* genes are rearranged in various cancers [16,17]; *FOXA1* is mutated in prostate and breast tumors [18,19,20,21], while *FOXM1* has been shown to be a potent outcome predictor in various cancer types [22].

## 3. FOXO Family

**Figure 2:**
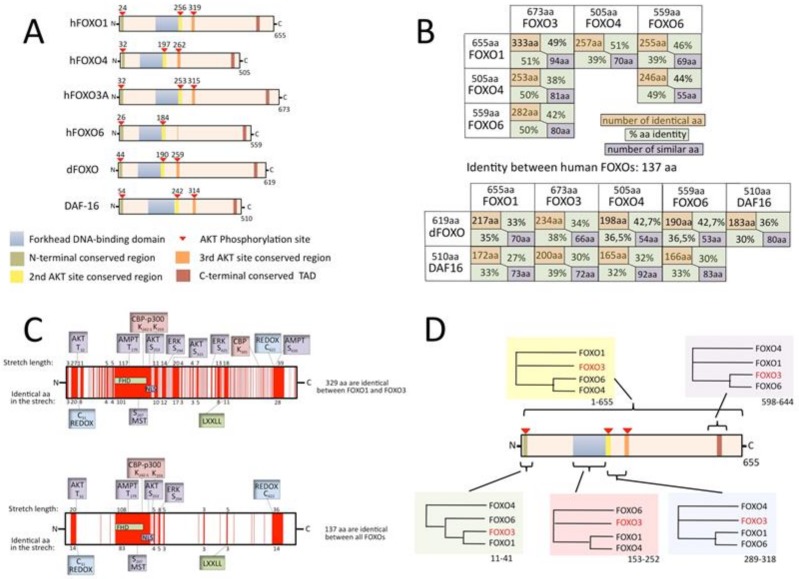
(**A**) Schematic representation of the four human FOXO members and their most important shared features; (**B**) identity in terms of amino acid number and percentage (related to each protein in the comparison) and number of similar amino acids. FOXOs are compared one to one (EMBOSS Needle, Matrix: EBLOSUM62, GAP penalty: 10.0, Extend penalty: 0.5 [33]). (**C**) Sequences that are identical in the two most similar proteins, FOXO1 and FOXO3 (top), and in all four human FOXOs (bottom) are depicted in red. (**D**) Phylogeny tree obtained with Clustal Omega when the indicated segments of the four human FOXO proteins are compared (the range of amino acid indicated below the boxes is relative to FOXO3 sequence).

In humans, five genes encode for FOXO transcription factors: *FOXO1*, *FOXO3*, *FOXO3B*, *FOXO4* and *FOXO6*. These are all regular genes, with the exception of the pseudogene *FOXO3B*, which has recently been shown to encode for a functional protein as well [23]. Bioinformatic analyses of DNA sequences have suggested that vertebrate *FOXO* genes originate from successive gene duplications [24]: the first leading to the *FOXO3/6* and *FOXO1/4* lineages, and two additional duplications leading to the four current genes. Figure 2A also shows the most important common features in the human paralogs, *C. elegans* DAF-16 and the *Drosophila* dFOXO proteins. Besides the very structured forkhead domain, most of FOXOs consist of intrinsically disordered regions [25]. These regions are characterized by an elevated functional versatility that is caused by a capacity to adopt different conformations and therefore interact with a number of different proteins [26,27]. One important interactor for FOXOs is the AKT Ser/Thr kinase [28,29,30,31]. The negative regulation of FOXO nuclear function by the INS/PI3K/AKT pathway is a hallmark of the FOXO family. Three AKT phosphorylation sites are highly conserved in evolution and are found in all six of the proteins represented in Figure 2A, with the exception of one phosphorylation site missing in FOXO6. The AKT phosphorylation site that is closest to the *N*-terminus lies in a short conserved region that is found in all of the proteins, the middle AKT site lies at the *C*-terminal extremity of the DNA-binding domain in a conserved nuclear localization signal sequence (NLS) and the third AKT site lies in a relatively short conserved sequence that serves as a nuclear export signal that is not found in FOXO6. Upon phosphorylation by AKT, FOXOs translocate to the cytoplasm with the exception of FOXO6, which, in most cases, does not follow this rule, as it lacks a nuclear export signal [32]. Many other post-translational modifications, such as non-AKT phosphorylation, acetylation, ubiquitination, methylation and cysteine oxidation, influence the cellular localization of FOXOs, as well as their DNA–binding, their transactivation and their binding to other proteins. These characteristics are differently conserved in FOXOs and have often not been functionally tested in all members.

### 3.1. Sequence Homology

After gene duplication, one of the paralogs is thought to mutate rapidly in order to obtain new functionality and be maintained (if unchanged, there would not be pressure to keep it, as its loss would not give a phenotype). This process is discussed at the end of this review. I decided to examine the proteins one by one, at the level of amino-acid identity, which is the most stringent way to compare conservation. The table in Figure 2B shows the number of identical amino acids and the percentage of identity (the percentage relative to each of the compared paralogs is shown to correct for differences in length). It appears that FOXO3 and FOXO1 share the most identity in terms of amino acid numbers (representing about 50% of their relative size). However, relative to its size, 50% of the FOXO4 sequence is identical to those of all three of the other FOXOs. FOXO6, which is considered to be the most divergent and quickly evolving [24] of the FOXOs, shares the most identity with FOXO3, substantially more so than with FOXO1 and FOXO4. When comparing human FOXOs with *C. elegans* and *Drosophila* FOXOs, we see that FOXO3 is the closest, while comparisons between Daf16 and dFOXO reveal that they have a lower identity rate, which may partly be due to differences in the generation numbers of the organisms. When all human FOXOs are compared (Clustal Omega 1.2.4 alignment [33]), the numbers fall: 137 identical amino acids and 78 similar amino acids (representation in Figure 2C). A possible explanation for this is that the FOXOs evolved in a way that kept specific areas closer to being identical in two or three paralogs, while other regions kept identity with the remaining paralog, so that overall identity between the four paralogs drops to a low level. I therefore decided to align the four human FOXO protein sequences in different manners, either using the entire protein sequences or specific parts of them. The outcome was evaluated by using the phylogeny tree provided by (Clustal Omega 1.2.4 alignment [33]). Figure 2D shows that phylogeny is not homogeneous along the protein sequence, meaning that some functionalities of a given FOXO are more shared with one paralog and others functionalities with another.

In conclusion, the average identity in human FOXOs is very high (around 50% relative to the shorter paralog). The forkhead domain is an important part of total identity, implying that average identity is lower in the rest of the protein. Furthermore, FOXOs contain high percentages of intrinsically disordered regions that are usually less conserved [25]. More detailed studies on sequence comparison are required in association with protein-structure analyses and protein-structure-based predictions of protein–protein interactions to explore these ideas. The determination of FOXO functional similarities and divergences may be useful when developing drugs to target FOXOs in cancer or other disease and to aid the understanding of the effect of polymorphism found in the population. The FOXO3B protein [23], which the product of a *FOXO* pseudogene, will not be treated in this review, because it is structurally quite different, with an 85-amino-acid extension at the *N*-terminus and a truncated non-functional forkhead domain with a stop codon in the mid-forkhead domain. The protein is permanently located in the cytoplasm.

### 3.2. FOXO Function

FOXO function is often described as and considered to be a collective family function. This is because they are often co-expressed, show co-regulation of their PMTs, have similar effects on reporter expression in vitro and have been shown, in some instances, to have additive, redundant or even synergistic effects in vivo. However, studies have often looked at one particular paralog, without studying the others in the same context. Furthermore, in some cases, double or triple conditional knockouts were compared to wild types, so that the effect of a single knockout in the exact same context remains unknown [34,35].

FOXOs control tissue homeostasis and are particularly involved in metabolic adaptation to nutrient intake (glucose and lipid metabolism), autophagy, protection from oxidative stress, aging, cell growth, apoptosis and cell-differentiation (reviewed in [36,37,38,39,40,41,42]).

FOXOs play a role in cancer [43], diabetes [44,45] and longevity [46,47], as well as other disease-related processes.

If FOXOs have a lot in common, there is no doubt that they have specific function as revealed by the difference in the outcomes of their respective knockouts in mice [48,49,50]. However, we must keep in mind that the phenotype of knockout mice for a single FOXO member may be milder than expected, because remaining FOXO family members may compensate for the missing knocked-out member. Furthermore, the different phenotype observed among different single FOXO member knockout may be due to the deletion of a common function shared by the FOXOs but that is expressed differently in the organism (specific paralog expression, see below). Finally, FOXOs regulate each other’s expression, as described below. All of these factors make it difficult to draw simple conclusions about the precise role of one specific FOXO paralog.

**Figure 3:**
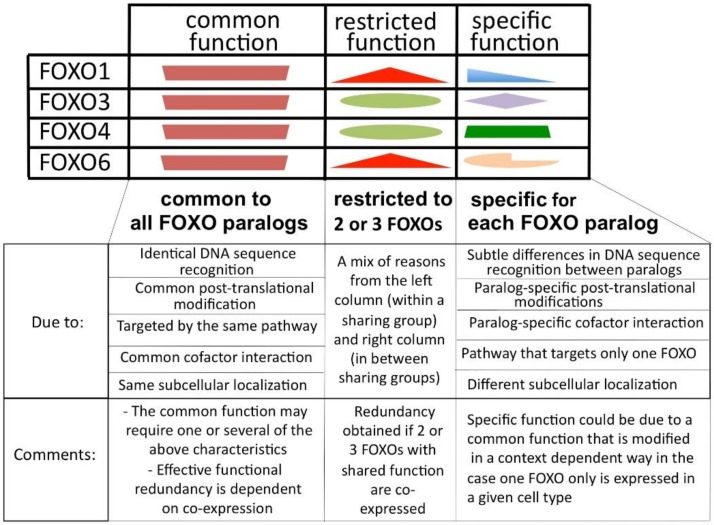
Possible family-related functional characteristics that could coexist in each of the paralog proteins. The space and location dedicated to the different functions in the table are not proportional to their importance or related to their location in the proteins.

Figure 3 schematically displays the different theoretical FOXO functions characterized in relation to their uniqueness among FOXO members: common functions that are shared by all members (left column), restricted functions shared by two or three members (central column) and paralog-specific function when present in only one of the paralogs (right column). When at least two FOXOs are expressed in the same cell, then the common function is then present at least twice and can be defined as functionally redundant.

The representation in Figure 3 is an oversimplification, but it helps in the reasoning that I will do later in the review. The notion of function in the table is relative to the protein, and it may be difficult, if not impossible, to ever test with precision what common, restricted or specific functions are. One could take the example of FOXO1’s role in the endothelium. The function that FOXO1 exerts in coupling metabolic activities and growth activities [51] could be classified as a specific function because a strong phenotype is seen after the deletion of FOXO1. However, it is possible, that another FOXO paralog could be able to rescue the absence of FOXO1 in this experimental setting and that the paralog-specific importance of FOXO1 is not due to its protein characteristics but to its expression. It is therefore difficult to give concrete examples of the different categories of functions of the FOXOs at the moment.

#### Redundancy in Cell Growth, Apoptosis and Metabolism

FOXOs have long been suspected of being tumor suppressors for two main reasons: because they are negatively regulated by a growth-inducing pathway (the PI3K/AKT pathway often activated in cancer) and because FOXOs have a pro-apoptotic and cell-growth inhibitory role. This expectation was confirmed in the seminal publication by Paik et al., which showed that the simultaneous deletion of FOXO1, FOXO3 and FOXO4 in mice gave rise to tissue-specific tumors [52]. The fact that tumors were not obtained after single or double FOXO-member knockout indicated the redundant functions of FOXOs as tumor suppressors, a view that was indirectly confirmed by the lack of mutations found in FOXOs in human cancer. Independent mutations in three FOXO genes, with no positive selection in terms of growth or survival after one or two hits (no driver effect), has very little probability to occur. On the other hand, rearrangements of FOXOs have been found: MLL-FOXO3 in leukemia [17] and PAX3-FOXO1 in rhabdomyosarcoma [16]. In both cases, the fusion proteins were shown to interfere with FOXO function, and were thus able to affect several FOXOs at once [53,54].

Another way to collectively inactivate the nuclear function of the FOXOs is by inducing the PI3K-AKT pathway [55,56]. This pathway is very often permanently activated in cancer by mutations that inactivate PTEN [57] or mutations that activate PI3K [58]. The pathway targets many effector proteins besides FOXOs, so it is therefore difficult to assess the exact contribution of FOXO in tumorigenesis. The view that FOXOs are general tumor suppressors has recently been challenged, and a more context-dependent view of the role FOXO in cancer has emerged [43].

FOXOs also have redundant functions in glucose metabolism and, more generally, in diet response. The simultaneous deletion of FOXO1 and FOXO3 in the liver is required to observe hypoglycemia and hyperlipidemia in mice [59,60,61]. Increased autophagy and muscle mass loss has been observed after the deletion of either the muscle insulin-receptor or IGF1-receptor, and is rescued by the loss of FOXO1, FOXO3 and FOXO4, but not if single FOXOs are deleted [62], which indicates a redundant role in autophagy. It has been described that FOXO1 and FOXO3 have overlapping roles in chondrocytes [63], and in the inhibition of myoblast differentiation [64]. For historical reasons, there has been a delay in the study of FOXO6 [65]. Furthermore, deleting FOXO6 in the cancer and metabolism models described above may prove to be interesting.

### 3.3. FOXO Expression

**Figure 4:**
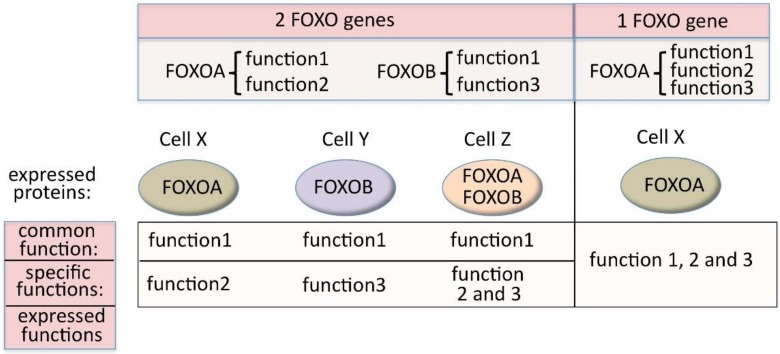
Possibility to express common functions in all cell types and specific functions in diverse cell types (left side) that would not be possible with the existence of a single FOXO gene (right side).

Human *FOXO* genes lie on different chromosomes, and therefore have autonomous expression and expression regulation due to their individual chromatin environments, gene promoters, enhancers and regulatory regions (regulated by miRNAs, for example) [66]. This means that, if we consider the different functionalities indicated in Figure 3, the expression and the regulation of the expression of the “common function” has broadened significantly. At the same time, the fact that there are several genes allows the separation in terms of expression of functionalities that are distinct between paralogs. This would not be possible if these were all carried by one gene (Figure 4).

FOXOs have a rather broad expression in the organism, especially FOXO1 and FOXO3, which are often co-expressed, leading to functional redundancy for a common function.

If FOXOs have autonomous expression, as seen above, then they are not independent, because of this. FOXOs regulate the expression of their paralogs and also their own expression [67,68,69,70,71]. This is another characteristic of functional family cooperation and is certainly required for their synchronized action as a group of proteins.

### 3.4. Mechanism of Action

Many signaling pathways regulate the subcellular localization of FOXOs. In this review, I principally consider the role that FOXOs exert in the nucleus, but FOXOs have also been found to act in the cytoplasm, where they regulate, for example, autophagy [72,73] and ERK activity [74]. FOXOs were also found in the mitochondria [75,76,77].

**Figure 5:**
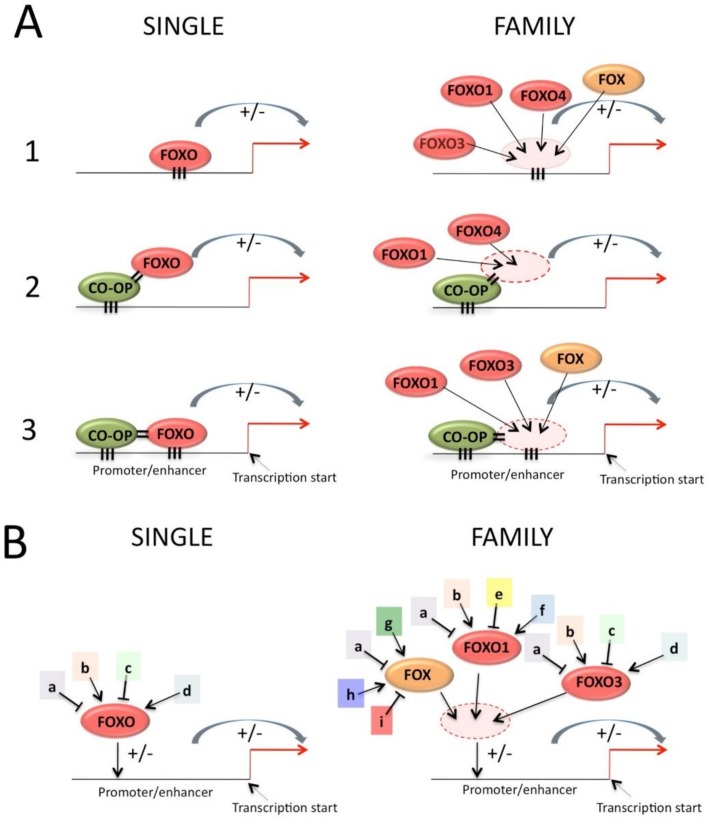
(**A**) Left side: simplified view of the major different modes of action of FOXOs: 1. Direct DNA-contact, 2. Indirect binding, through another protein making DNA-contact, 3. Pairwise binding, with both proteins making DNA-contact. Right side: Family effect for the different mode of action. (**B**) Left side: different pathways can influence activity of FOXOs whatever mode of action it uses. Right side: possible family effects that greatly enhance the possibilities of regulation. All the examples are arbitrary and do not reflect real situations.

The mode of action determines how similarity in a protein family is exploited functionally. FOXO can regulate transcription in a number of ways in regard to DNA binding, as shown in Figure 5:

1. Binding to DNA in an autonomous way. The DNA-binding capacity can be regulated by post transcriptional modification, such as phosphorylation (reviewed in [78]) or acetylation [79,80]. Acetylation via KDM5 interaction may have promoter-specific effects, so that not all FOXO-dependent promoters are affected [81];
2. Binding to another DNA-binding protein without FOXO DNA-binding [82,83,84,85,86];
3. Pairwise binding to DNA in which the binding of FOXO to DNA is dependent on the interaction with another protein that itself binds a nearby DNA sequence, such as for FOXO and SMAD [87]. This mode of action establishes a certain degree of cooperation that can result in a synergistic effect on transcription [88]. Changes in protein or DNA conformation after the first interaction can increase the binding affinity of the second interactor so that pairwise binding has more than an additive effect on transcription. These conformational changes may explain why the pairwise binding mode often alters the DNA binding specificity of the transcription factors involved [11]. Finally, the fact that two binding sites need to be nearby and at a precise spacing creates the basis for the establishment of elevated selectivity (because the random presence of single transcription factor binding site in the genome is relatively high). Pairing partners could belong to the same family or subfamily. This is discussed in the DNA-binding section below.

Transcription factors only bind a subset of their recognition motifs that are present in the genome. One of the reasons for this is the local chromatin structure, which may or may not permit easy access to DNA. Accessibility is epigenetically regulated through histone modification, DNA methylation and chromatin remodulation. Chromatin immunoprecipitation coupled with high-throughput DNA sequencing (ChIP-seq) allows the genome-wide occupancy of a given factor on chromatin to be determined. ChIP-seq analyses have revealed that occupancy does not necessarily indicate the presence of high-affinity binding sites for a giving transcription factor. It is often low-affinity binding sites that are occupied, suggesting that cooperation in binding with additional factors is common. To make things even more complicated, the binding of a given transcription factor on regulatory sequences does not guarantee an effect on transcription [89].

## 4. Family Effect

**Figure 6:**
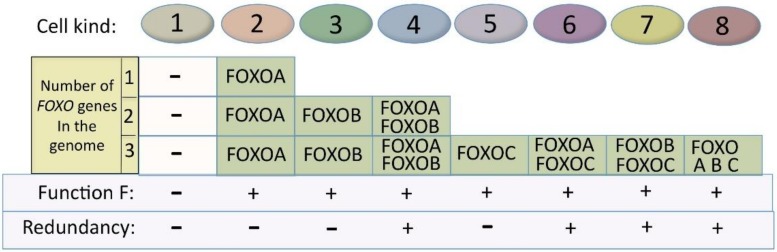
Increase in *FOXO* gene numbers increases the number of cell kinds (that can include several cell types) that express Function F and the number of cell kinds redundant for it.

The Family effect can be exerted in two ways: (1) same cell effect, due mainly to the common DNA-recognition specificity, among family members, that creates functional overlap. The benefit of this effect is that a single DNA element increases its regulatory potential by being hit by more pathways and effector proteins (Figure 5, right column). (2) A transversal effect across cell types or tissues, which enables the extension of the repertoire of cells that express a common function while expressing different paralog-specific functions (Figure 6). This may be one of the mechanisms by which more complexity in organisms is achieved by creating new functionality that is expressed in new cell types, while common essential functions are preserved. These two families effect mechanisms will be discussed below.

### 4.1. The Family Effect Enhances Functionality within a Cell

This effect is mainly caused by the DNA-recognition specificity that is shared by FOXOs family members, but also by other FOX proteins.

The crystal and solution NMR structures of several FOX protein-DNA complexes have revealed that the highly conserved residues of helix H3 bind the major groove of DNA and make the majority of the base contacts. In different FOX members, wing residues can make additional specific DNA contacts. An additional alpha-helix is present in some FOX proteins (including all FOXOs), and the FOXOs subgroup share a common 5 amino-acid insertion that changes the length of the loop between helix H2 and H3 [90]. These few examples of common and divergent characteristics explain why FOX proteins can recognize similar sequences, the core being RYAAAYA (R = G/A and Y = C/T), but show variability in recognition at the extremity of the core.

Within each subgroup, DNA-sequence recognition is very similar if not identical. Differences in specificity can vary between subfamilies.

Besides the fact that different FOX family members can recognize very similar sequences [91], the idea has emerged that FOX members from different family can possess shared functions, as well as restricted function: FOXA1, FOXG1 and FOXD3 can each recognize specific sequences that are not recognized by the other two members. Some variant DNA sequences can, however, be recognized by two or even all three FOX proteins [92]. This concept of “individual and shared” that emerged from this early in vitro-binding study has been confirmed by more recent ChIP-seq and functional studies.

In muscles, FOXK1 and FOXK2 repress a set of genes involved in autophagy that overlap with the one induced by FOXO3, so that, in starved conditions, FOXO3 is found on promoters were FOXK was found in non-starved conditions [93]. In cardiomyocytes, FOXM1 and FOXO1 show antagonist effects on cell growth and IGF1 expression that depend on an identical binding element [94].

In hepatocytes, 58% of DNA sites occupied by FOXO1 are also occupied by FOXA2, with carboxylic acids, lipids, steroids and vitamin A metabolism being shared functions, while FOXO1 has a specific function in DNA metabolism and FOXA2 in hepatic development [95]. A similar study compared the chromatin binding of FOXK2, FOXO3 and FOXJ3 [96] and identified several pools of genomic regulatory fragments that were able to bind more than one of the three factors: different pools recognized FOXK2 and FOXO3; FOXK2 and FOXJ3; FOXO3 and FOXJ3; and FOXK2, FOXO3 and FOXJ3.

The mechanisms at play when several proteins regulate transcription through identical cis-acting elements are not known with precision. The interplay of factors may follow different rules, depending on the binding site and the proteins involved: direct competition, exclusion and partial site occupancy, but also cooperation. A great deal must still be learned about binding kinetics, especially in the context of chromatin. Protein–DNA association is concentration-dependent, and when more than one factor is recognizing the sequence, the different affinities for the particular sequence, the diverse concentrations of the factors, their capacity to adapt to the local chromatin state and the presence of co-factors will decide the outcome. Moreover, the dissociation of the DNA–protein complex is relevant. Dissociation was long thought to be spontaneous and independent of concentration. However, recent data have contradicted this view. Higher concentrations of a given transcription factor increase its own off rate, which is a so-called “facilitated dissociation”, shown to occur with dimeric and also with monomeric DNA-binding proteins [97,98]. The exact molecular mechanism of facilitated dissociation is not known. It would be interesting to discover whether such induced dissociation can occur between FOXO family members or even between FOXO and other FOX superfamily members. A partial occupancy mechanism has been shown to occur with FOXK2, FOXO3 and FOXJ3 [96]: The addition of one factor increases its own binding without a decrease in the binding of the other. It is possible that the facilitated dissociation, described above, plays a role in such a mechanism: One factor is able to dissociate itself from DNA but is less effective to dissociate the other co-expressed factor binding to the same site (or vice versa). It would be interesting to check these possibilities.

Cooperation between two family members is another possible contribution to the family effect. As mentioned previously, many binding sites are not accessible to transcription factors, because the chromosomal region in which they lie in is in an unfavorable chromatin state.

The peculiar characteristics of certain transcription factors enable them to bind DNA/nucleosomes that are located in unfavorable chromatin regions and to initiate a process of chromatin activation. These phenomena are often linked with changes in cell-differentiation stages. A closed chromatin state is part of a mechanism of gene silencing and needs to be relieved to permit the expression of genes that are important for the process of differentiation itself and/or for the function of the differentiated cell. The transcription factors that can perform these tasks have been therefore been called pioneer factors [99].

A FOX member is one of the prototypes for pioneer factors. FOXA1 (HNF3) is able to bind histones and to open compacted chromatin [100].

Transcription factors that possess pioneer factor ability start the process of chromatin remodeling, enabling further binding of transcription factors that do not have this characteristic. This collaboration may happen between FOXA members and FOXO members. FOXA members are essential for the embryonic initiation of hepatic differentiation, but not for the maintenance of nucleosome organization later on. FOXOs, and in particular, FOXO1 are important in many hepatic functions and metabolism. The abovementioned results [95], which describe the co-binding of FOXA and FOXO1, are significant in this sense, although they were obtained in differentiated hepatocytes. Another type of cooperation can be found between FOXD3 and FOXOA1 on the Alb promoter. FOXD3 is expressed in ES cells and binds to the Alb enhancer, marking it for further binding and action by FOXOA1 and FOXA2 during gastrulation [101,102]. Much must still be learned, and new technologies will prove useful in understanding the mechanisms involved [103]. FOXOs have the capacity to interact with chromatin and this was the subject of a review [104].

Besides the recognition of the core FOX recognition sequence by many FOX proteins, other types of sequence recognitions have been reported within FOX proteins. I will mention some here (Section 4.1.1), because some reports are recent and show that our knowledge about DNA-binding is far from definitive. One of the reasons for alternative binding specificity is the pairwise mode of binding of the FOX protein, referred to below, in Section 4.1.2. This mode could be another way in which the “family effect” is exerted. Finally, the conservation, during evolution, of binding specificity is addressed in the Section 4.1.3.

#### 4.1.1. Non-Canonical Binding

FOXN1 and FOXN4 recognize the 5bp (GACGC), which is very different from the FOX-consensus sequence [105,106,107,108,109]. FOXN2, FOXN3 and FOXM1 possess bi-specificity: They recognize both the FOX core sequence and the (GACGC) sequence for FOXN3 and FOXN4 and an inverted repeat for FOXM1 [106]. A non-canonical functional binding site has also been reported for FOXO1 [110].

When transcription factors bind to nucleosome, their DNA-binding specificity can change. This has been shown for the pioneer factor FOXA1 [103].

#### 4.1.2. Pairwise Binding

Fox proteins bind DNA as monomers, unlike the b-ZIP or the bHLH transcription factors that require homo- or hetero-dimerization to function. However, site selection experiments have shown evidence of a dimeric mode of binding, even for proteins acting as monomers [111], including FOX family members [106,112]. These experiments performed with a high concentration of purified and tagged proteins could carry artifacts, but the very precise spacing and orientation of the binding sequences selected (overlapping and inverted for FOXM) support the fact that very ordered complexes are formed and might be functional. A decisive recent publication supports this view: Functional DNA-mediated FOXA1 homodimer formation was identified on a palindromic DNA element in the context of chromatin [113].

Do other FOX form DNA-mediated homodimers or even FOX hetero-dimers? Some evidence that these kinds of interactions exist come from proteomic analysis, using affinity selection and mass spectrometry [114]. Furthermore, synergy has been shown to occur between FOXOs [59], although the mechanism is unknown.

#### 4.1.3. Conservation of DNA-Binding Specificity during Evolution

During evolution, the conservation of binding specificity is very strong, and *C. elegans* DAF-16 and mouse FOXOs give identical core binding sequence (TTGTTTAC) [115]. More generally, Nitta et al. [116] have compared the binding specificity of over 200 TF in *Drosophila*, mice and humans. They found remarkable conservation between orthologs (over 600 million years), and changes were often linked to the appearance of new functions in new cell types that were not found in *Drosophila*. Remarkably, human FOXO3 can functionally substitute DAF16 in *C. elegans*, although only partially [117]. The conservation of binding specificity of the DNA binding domain contrasts the poor conservation of their targets, namely the motifs to which they bind in promoters and enhancers of target genes [6] and the poor conservation of the protein sequences outside of the DNA-binding domain which serve as a functional regulator of it. It is intuitive to think that it is easier to create new functionality during evolution by moving small DNA motifs around in the genome or by modifying the regulatory sequences of a DNA binding domain, rather than by changing the DNA-binding domain, which is a quite complex structure, of around 100 amino-acids, that is required for specific DNA-recognition. In addition, and this is one of the topics of this review, the conservation of DNA-recognition specificity in a group of proteins can have functional advantages.

### 4.2. The Family Effect Facilitates the Creation of Functionality in New Cell Types during Evolution

The principle is shown in Figure 6. For simplification, I use the following theoretical example: FOXO is regulating a fundamental process that is common to many cell types and is symbolized here by Function F. If one *FOXO* gene is present in the genome, there will be two possible kinds of cells (I use the term “kind”, because one kind could include a number of different cell types), in regard to Function F: kind 1, which is negative for it, and kind 2, which expresses it. If two *FOXO* genes (both with Function F) are present, then we will have four situations, as shown in Figure 6, with the number of cell kinds (that can include different cell types) expressing Function F rising from one (with one *FOXO* gene) to three (with two *FOXO* genes). When the *FOXO* gene number rises to three or four, then the number of cell kinds that are positive for Function F rises to 7 and 11, respectively.

Figure 6 also provides somewhat of an idea of the timely expansion of new cell kinds (or the cell types within).

More complex organisms mean more specialized tissues and organs with many more cell types being required. In our example, the broadening of the expression of Function F, due to increase in *FOXO* genes (each having individual chromosome localizations, gene promoters, enhancers and additional regulatory regions), enables and participates in the creation of new cell types. In Figure 6, we see that having three *FOXO* genes, creates seven different situations with Function F expression. With seven situations of expression (compared to one with one gene), there are many more chances for Function F expression to adapt and coevolve with the change in global expression pattern required for the establishment of new cell type phenotypes in more complex organisms.

Furthermore, apart from Function F that remains, new functions can be added or lost on the different *FOXO* paralog genes. An example of this is the evolutionary acquisition of functional cysteines [118]. These new gene functions (paralog-specific) can actively participate in the creation of new cell types, while the common function remains. We have already seen, in Figure 3, the advantage of having specific functions spread on several genes. Another consequence of having two or more *FOXO* genes (as in Figure 6), with both common and paralog-specific functional parts, is that some cell kinds will be redundant for Function F. With two *FOXO* genes, one cell kind (number 4) will be redundant for Function F. With three FOXO genes, four cell kinds (numbers 4, 6, 7 and 8) will be redundant for Function F. Redundancy is discussed later, in the evolution section.

## 5. Back to *C. elegans*

I started this review with a comparison between the five genes present in humans and the single *FOXO* ortolog *DAF-16* gene in *C. elegans*, which is famous for being required for lifespan extension in insulin receptor (DAF2) mutants [119,120,121]. More recent studies have proven that the newest discovered isoform d/f of the DAF16 protein can cooperate and synergize [122] with the older identified isoform a [117] in influencing lifespan. This very much resembles what I have described, herein, for the human proteins, although the precise mechanisms are still unknown.

Isoform d/f [122] is expressed from a different promoter. It possesses a different *N*-terminus responsible for its different nucleo-cytoplasmic distribution compared to isoform a (due to differential phosphorylation by AKT1 and AKT2). The cooperation between the two DAF16 isoforms in life-span extension is dependent on both the different expression pattern and the different sensitivity levels displayed by the two isoforms toward AKT kinases [122]. The remarkable structural and functional conservation of the Insulin receptor-FOXO pathway, between human and *C. elegans*, was confirmed by the finding that, in humans, like in *C. elegans* and *Drosophila*, FOXO might be involved in age regulation. An *FOXO3* genetic variant correlated with increased longevity in humans [123,124]. The analogy between cooperation of splicing variants of *FOXO* in *C. elegans* and gene variants of *FOXO* in humans is striking and may deserve more attention. Could it be that besides an impressive conservation in function and structure, the FOXOs from humans and DAF-16 from *C. elegans* also share the functional family effect described herein?

## 6. Evolutionary Considerations

As discussed above, FOXOs are partly redundant in their function, with many of the characteristics of this redundancy still to be defined.

Maintenance of genetic redundancy is not rare, and for some genes, it has been shown to extend over a period of billions of years of natural selection [125]. However, the mechanisms of this maintenance are difficult to explain, as its loss would, in theory, not show a phenotype.

After gene duplication, one of the two genes retains the initial function, meaning that there is no immediate selective pressure on the second one to be kept. Preservation theories include rapid mutations leading to new functions and changes in expression, so that gene dosage renders the expression of the two paralogs necessary [126]. It is also possible that excess initial expression, or differential expression due to newly acquired cis-acting regulatory sequences in the new paralog, initially represents a selective advantage so that both genes are maintained.

Once the paralog is established in its new function, what are the forces maintaining redundancy? Redundancy is expected to be transparent to selection pressure, and therefore, it is not maintained. The FOXOs are interesting in this regard and highlight the fact that the question of genetic redundancy should be addressed one gene or protein at a time, because the reasons for it and advantages that it represents are very much linked to the function and mechanism of action of the individual protein.

One of the theories developed to explain genetic redundancy could be applied to the FOXO family: the piggyback model [125], which refers to older theoretical work [127]. In this model, the redundant function overlaps (structurally in the protein) with the specific function. Germline mutations that alter the redundant function (no phenotype) would also affect the non-redundant function and therefore affect the phenotype. Natural selection would not permit such mutations and the common function would therefore be preserved. I have assumed that each FOXO contains a functional part that is shared with other paralogs and a part that is specific to itself (Figure 3). It is possible that these two kinds of functions are dependent on common parts of the protein and cannot be separated physically one from the other. They would therefore depend on each other for their preservation. This model is satisfactory for unicellular organisms or multicellular organisms in which the paralogs that are responsible for redundancy are always expressed together and never individually.

For multicellular organisms an additional mechanism applies. As seen in Figure 6, some cell kinds express only one of the FOXOs. Therefore, the germline loss of common function of this precise FOXO would have a negative effect for this cell kind and be selected against. It is the expression of the paralog as only paralog in some cells that actively selects for redundancy maintenance, and it is the need for the expression of several paralogs (due to the need for several paralog-specific function) in the same cell that makes redundancy revealed. It is therefore the alternation between single and multiple FOXO paralog expression that retains redundancy in association with a piggyback mechanism. This alternation, if occurring, during development would have an even greater selective effect.

Concerning the tumor-suppressive role of FOXOs, an audacious idea comes to mind when associating Figure 6 with the tumors that are obtained only if three FOXOs were simultaneously deleted [52]: deleting FOXOA, B and C would give tumors that originate from cells that express FOXO A, B and C, because other cells that only express one FOXO would have kept or enriched their tumor suppressive potential by other mechanism during evolution (and therefore single FOXO deletion would not give tumors). On the other hand, cells that are well covered by tumor-suppressive activity, because they express several FOXOs, would be subject to tumors (and in a cell specific way) after experimentally deleting three FOXOs (as observed in [52], an event that has almost no chance of occurring naturally.

The points discussed above weaken the theory that the establishment of redundancy, by co-expressing several FOXOs with a common function, was selected during evolution, to protect the organism from somatic mutations in the FOXO genes. It rather strengthens the possibility that redundancy is the result of other selective needs and is a by-product of it.

More experimental data are required to answer some questions about the entangled functionality of FOXO paralog proteins, and new technologies will greatly help to resolve these questions in the future [128].

The fate of duplication has been tested in models, such as yeast [129,130,131], and progress to understand the forces of billions of years of natural selection induces stimulating debate [132].

## 7. Conclusions

Although FOX proteins members, and even more, FOXO family members within their subfamily, have acquired new functions compared to their paralogs over the course of evolution, they have kept strong relations that enable mechanisms of collaboration, interference, exclusion and redundancy that enhance functionality and regulation potential. Most of these effects are conveyed through the common forkhead domain, exploiting in the paralogs the coexistence of shared and restricted DNA-binding recognition, and the coexistence of both similar and different activity regulation. The existence of several *FOXO* genes provides the possibility of the extended expression of their common functions and the segregated expression of the paralog-specific functionalities that would otherwise be expressed together if present on a single gene (under the condition that this would be possible at all). The FOXOs may represent an excellent model for the study of the maintenance of functional redundancy in evolution, a phenomenon that is not easy to explain, because of an apparent lack of selection for it. One possible mechanism is that the common function is preserved on paralogs (at least two to have redundancy) during evolution because required in cells where one FOXO is expressed. When now two or more FOXOs are expressed (because of the need for several paralog-specific functions), the redundancy is created. What are the common functions shared by FOXOs? Regulation of cell growth and apoptosis, protection from particular stresses, and cell metabolism regulation perhaps all of these ultimately influence the aging process.

For the moment, too little is known about specific and common functions within the FOXO family. Paralogs are mostly studied individually. For example, precise protein expression and localization information, as well as the sensitivity to diverse post-translational modifications is rarely studied for all the paralogs in an identical given situation. Functional comparisons between paralogs coupled with sequence comparisons might provide a tool that can address mechanistic questions but also understand the forces that molded, through evolution, the FOXOs as we know them now.
